# Genome‐Wide 3′‐UTR Single Nucleotide Polymorphism Association Study Identifies Significant Prostate Cancer Risk‐Associated Functional Loci at 8p21.2 in Chinese Population

**DOI:** 10.1002/advs.202201420

**Published:** 2022-06-17

**Authors:** Ning Zhang, Da Huang, Guangliang Jiang, Siteng Chen, Xiaohao Ruan, Haitao Chen, Jingyi Huang, Ao Liu, Wenhui Zhang, Xiaoling Lin, Yishuo Wu, Qin Zhang, Jing Li, James Hok‐Leung Tsu, Gong‐Hong Wei, Rong Na

**Affiliations:** ^1^ Department of Urology, Ruijin Hospital Shanghai Jiao Tong University School of Medicine Shanghai 200025 China; ^2^ Department of Urology, Renji Hospital Shanghai Jiao Tong University School of Medicine Shanghai 200080 China; ^3^ School of Public Health Shenzhen Sun Yat‐sen University Guangzhou 510006 China; ^4^ Department of Urology, Changhai Hospital Second Military Medical University Shanghai 200433 China; ^5^ Department of Urology, Huashan Hospital Fudan University Shanghai 200040 China; ^6^ Biocenter Oulu, Faculty of Biochemistry and Molecular Medicine University of Oulu Oulu 90014 Finland; ^7^ Department of Bioinformatics, Center for Translational Medicine Second Military Medical University Shanghai 200433 China; ^8^ Division of Urology, Department of Surgery, Queen Mary Hospital The University of Hong Kong Hong Kong China; ^9^ MOE Key Laboratory of Metabolism and Molecular Medicine & Department of Biochemistry and Molecular Biology, School of Basic Medical Sciences, and Fudan University Shanghai Cancer Center Shanghai Medical College of Fudan University Shanghai 200032 China

**Keywords:** 3′‐UTR, microRNA, NKX3‐1, prostate cancer, single nucleotide polymorphism

## Abstract

MicroRNAs (miRNAs) are involved in the regulation of gene expression via incomplete base pairing to sequence motifs at the three prime untranslated regions (3′‐UTRs) of mRNAs and play critical roles in the etiology of cancers. Single nucleotide polymorphisms (SNPs) in the 3′‐UTR miRNA‐binding regions may influence the miRNA affinity. However, this biological mechanism in prostate cancer (PCa) remains unclear. Here, a three‐stage genome‐wide association study of 3′‐UTR SNPs (*n*=33 117) is performed in 5515 Chinese men. Three genome‐wide significant variants are discovered at 8p21.2 (rs1567669, rs4872176, and rs4872177), which are all located in a linkage disequilibrium region of the *NKX3‐1* gene. Phenome‐wide association analysis using the FinnGen data reveals a specific association of rs1567669 with PCa over 2,264 disease endpoints. Expression quantitative trait locus analyses based on both Chinese PCa cohort and the GTEx database show that risk alleles of these SNPs are significantly associated with low expression of *NKX3‐1*. Based on the MirSNP database, dual‐luciferase reporter assays show that risk alleles of these SNPs downregulate the expression of *NKX3‐1* via increased miRNA binding. These results indicate that the SNPs at the 3′‐UTR of *NKX3‐1* significantly downregulate *NKX3‐1* expression by influencing the affinity of miRNA and increase the PCa risk.

## Graphical abstract

A genome‐wide three prime untranslated region (3′‐UTR) single nucleotide polymorphism (SNP) association study identifies 3 genome‐wide significant SNPs at 8p21.2, which are further confirmed by phenome‐wide association analysis, expression quantitative trait locus analyses, and dual‐luciferase reporter assays. The SNPs at the 3′‐UTRs of *NKX3‐1* significantly downregulate NKX3‐1 expression via increasing the affinity of targeted microRNA and might drive prostate cancer.

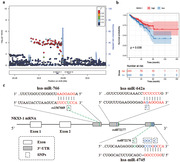

## 1 Introduction

With an estimated 1 414 259 new cases and 375 304 deaths worldwide in 2020, prostate cancer (PCa) has become one of the most common malignancies among men worldwide.^[^ ^1^ ^]^ Inherited risk is considered the major risk factor for PCa. Among men with a family history of PCa, the risk of PCa may increase by approximately twofold.^[^ ^2^, ^3^ ^]^ In addition to family history, more than 150 single nucleotide polymorphisms (SNPs) were found to be associated with PCa risk in Caucasians via genome‐wide association studies (GWAS).^[^ ^4^ ^]^ These SNPs were estimated to account for ≈33% of the inherited risk of PCa.^[^ ^5^ ^]^

To date, the inherited risk of PCa has been poorly studied in the Chinese population. Only one GWAS identifying 2 unique loci was reported by our group in 2012.^[^ ^6^ ^]^ Two additional risk‐associated loci were identified in the East Asian population via a large‐scale GWAS meta‐analysis of Chinese and Japanese populations.^[^ ^7^ ^]^ These SNPs can only explain a very small part of the inherited risk in the Chinese population. More importantly, the biological functions of these SNPs are poorly understood.

A previous study suggested that SNPs in microRNA (miRNA) binding sites of genes might contribute to PCa risk in Caucasians.^[^ ^8^ ^]^ These SNPs are located within the three prime untranslated regions (3′‐UTRs) of the target genes which may influence the affinity of certain miRNAs and regulate gene expression. Despite several tagging SNP association studies,^[^ ^9^, ^10^ ^]^ a comprehensive or genome‐wide association study between 3′‐UTR SNPs and PCa risk has never been reported in the Chinese population.

Therefore, to identify additional PCa risk‐associated SNPs and explain their biological functions, we conducted the present three‐stage genome‐wide 3′‐UTR SNP association study that evaluated the association between 33 117 3′‐UTR SNPs and PCa risk in ≈5500 Chinese men.

## 2 Results

This was a three‐stage genome‐wide 3′‐UTR SNP association study based on Chinese population. Three independent case‐control cohorts were evaluated in the present study. The demographic characteristics of the study population were described in Table S1, Supporting Information. Detailed information on the study population was published elsewhere.^[^ ^6^, ^11^ ^]^

**Figure 1:**
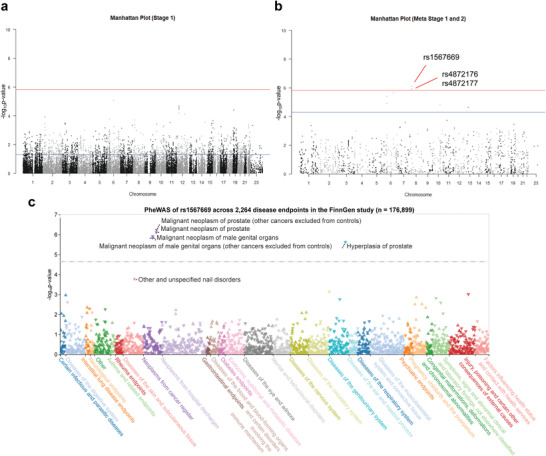
3′‐UTR‐GWAS and Phenome‐wide association analysis (PheWAS) results for prostate cancer. a) Manhattan plot of Stage 1 (genome‐wide 3′‐UTR SNP association study). Red line: Bonferroni correction significance level; blue line: *P* = 0.05. b) Manhattan plot of meta‐analysis (Stages 1 and 2). Red line: Bonferroni correction significance level; blue line: *P* = 5 × 10^−5^. c) PheWAS of the associations between rs1567669 and 2264 disease endpoints in the FinnGen study (*n* = 176 899). Significant Bonferroni corrected threshold was defined at *P* = 0.05/2264 = 2.21 × 10^−5^. The vertical axis shows the associated *P*‐values on the −log_10_ scale, and the horizontal axis indicates categories of disease endpoints.

A total of 1959 SNPs reached *P* < 0.05 in Stage 1 (**Figure** **1**a). None of the SNPs reached the genome‐wide significance level (*P* < 1.51 × 10^−6^). These SNPs were further evaluated in the Stage 2 confirmation study. The result of the meta‐analysis between Stage 1 and Stage 2 is shown in Figure 1b. We found that the 3′‐UTR SNPs rs4872177 (odds ratio [OR] = 1.32, *P* = 8.18 × 10^−7^), rs4872176 (OR = 1.31, *P* = 1.30 × 10^−6^), and rs1567669 (OR = 1.31, *P* = 1.21 × 10^−6^) at Chr8 reached a genome‐wide significance level (**Table** **1**). Seven additional risk‐associated SNPs at Chr6, Chr10, and Chr13 reached *P* < 5 × 10^−5^ (Table 1). These SNPs were further evaluated in Stage 3 confirmation. The SNPs (rs4872176, rs4872177, and rs1567669) located in 8p21.2 remained significantly associated with PCa and reached genome‐wide statistical significance in the meta‐analysis of the three stages (Table 1). Furthermore, we performed a phenome‐wide association analysis (PheWAS) in the FinnGen study^[^ ^12^ ^]^ (*n* = 176 899) and examined the associations between the three individual SNPs and 2264 disease endpoints. Intriguingly, we found the strongest PheWAS association of rs1567669 was with malignant neoplasm of prostate (4754 cases and 63 465 controls), suggesting that this SNP is associated with PCa risk in the different ethnic groups (Figure 1c). The Q‐Q plot of Stage 1 is presented in Figure S1, Supporting Information (*λ* = 1.069). All three SNPs were located in the linkage disequilibrium (LD, *R* ^2^ > 0.95) region of the *NKX3‐1* 3′‐UTR (Figure S2, Supporting Information).

**Table 1:** Results of 3‐stage association study between prostate cancer and miRNA binding site SNPs. (SNPs with *P* < 5 × 10^−5^ at meta‐analysis of Stage 1 and 2 were shown)

No.	SNP	Chr	Position	A1	Stage 1		Stage 2		Meta‐analysis of Stage 1 and 2^a)^			Stage 3		Meta‐analysis of three stages^a)^		
					OR (95%CI)	*P*	OR (95%CI)	*P*	OR	*P*	I^2^	OR (95%CI)	*P*	OR	*P*	I^2^
1	rs1059288	6	33 267 672	G	1.31 (1.14–1.50)	1.68E‐04	1.23 (1.06–1.43)	0.0059	1.27	3.82E‐06	0	1.05 (0.91–1.21)	0.53	1.19	0.008	58.93
2	rs1061783	6	33 282 628	T	1.27 (1.10–1.46)	9.44E‐04	1.25 (1.07–1.45)	0.0042	1.26	1.25E‐05	0	1.09 (0.94–1.27)	0.24	1.20	2.01E‐05	14.24
3	rs631089	6	117 252 672	A	0.81 (0.70–0.93)	0.0034	0.75 (0.64–0.87)	1.51E‐04	0.78	2.24E‐06	0	0.92 (0.79–1.07)	0.30	0.82	6.69E‐06	46.74
4	rs630695	6	117 252 759	G	0.81 (0.70–0.93)	0.0034	0.75 (0.64–0.87)	1.51E‐04	0.78	2.24E‐06	0	0.92 (0.79–1.07)	0.30	0.82	6.69E‐06	46.74
5	rs4872176	8	23 538 008	C	1.30 (1.12–1.51)	6.72E‐04	1.32 (1.13–1.54)	5.72E‐04	1.31	1.30E‐06	0	1.18 (1.01–1.37)	0.04	1.26	2.83E‐07	0
6	rs4872177	8	23 538 426	A	1.33 (1.15–1.55)	1.91E‐04	1.30 (1.11–1.52)	0.0012	1.32	8.18E‐07	0	1.15 (0.99–1.35)	0.07	1.26	3.98E‐07	0
7	rs1567669	8	23 538 533	A	1.31 (1.13–1.52)	4.00E‐04	1.30 (1.12–1.53)	8.88E‐04	1.31	1.21E‐06	0	1.19 (1.02–1.39)	0.03	1.27	1.51E‐07	0
8	rs12948	10	91 534 524	T	1.27 (1.09–1.47)	0.0023	1.30 (1.10–1.53)	0.0021	1.28	1.53E‐05	0	1.06 (0.91–1.24)	0.47	1.20	8.02E‐05	46.22
9	rs10982	10	91 534 527	A	1.27 (1.09–1.47)	0.0023	1.30 (1.10–1.53)	0.0021	1.28	1.53E‐05	0	1.01 (0.86–1.20)	0.87	1.19	0.025	61.79
10	rs7335649	13	43 364 859	G	0.78 (0.66–0.92)	0.0031	0.76 (0.63–0.91)	0.0025	0.77	2.35E‐05	0	0.99 (0.81–1.19)	0.92	0.83	0.027	59.93

Abbreviations: SNP, single nucleotide polymorphism; OR, odds ratio; 95%CI, 95% confidence interval.

^a)^ ORs and *P*‐values were estimated using random‐effect model if *I* ^2^>50

**Figure 2:**
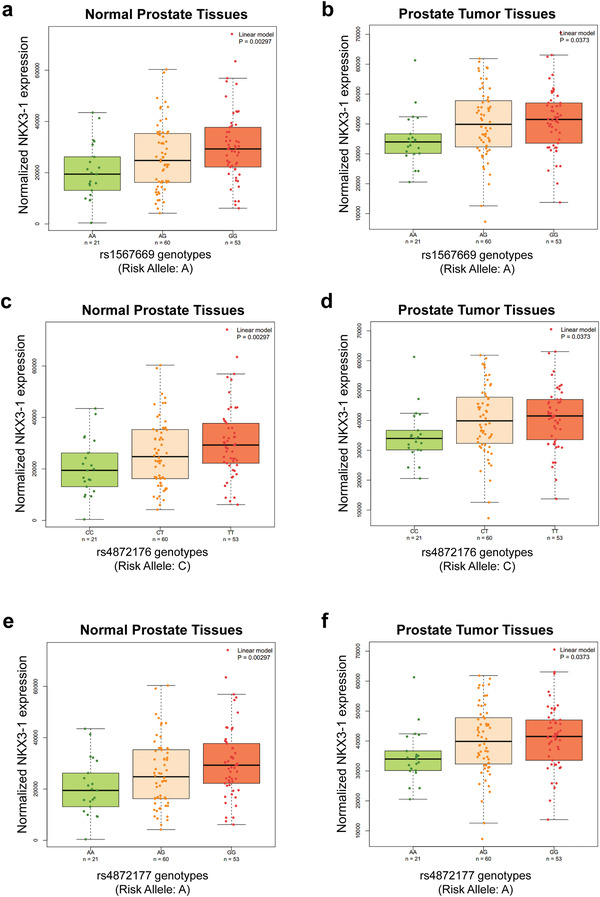
Cis‐eQTL analyses in the Chinese Prostate Cancer Genome and Epigenome Atlas (CPGEA) study.^[^ ^14^ ^]^ The normalized expression levels of *NKX3‐1* were significantly lower when carrying the risk alleles of the three target SNPs in both normal and tumor tissues of prostate (*N* = 134, all *P* < 0.05).

Expression quantitative trait locus (eQTL) analyses based on the Genotype‐Tissue Expression (GTEx) database^[^ ^13^ ^]^ showed that risk alleles of these SNPs were significantly associated with low expression of *NKX3‐1* in different normal tissues (all *P* < 1.00 × 10^−4^, Table S2, Supporting Information). These results were further confirmed in the Chinese Prostate Cancer Genome and Epigenome Atlas (CPGEA) prostate normal and tumor samples (*N* = 134).^[^ ^14^ ^]^ As shown in **Figure** **2**, the risk alleles of the significant SNPs were significantly associated with lower expression of *NKX3‐1* in both normal and tumor tissues of prostate in the Chinese population.

**Figure 3:**
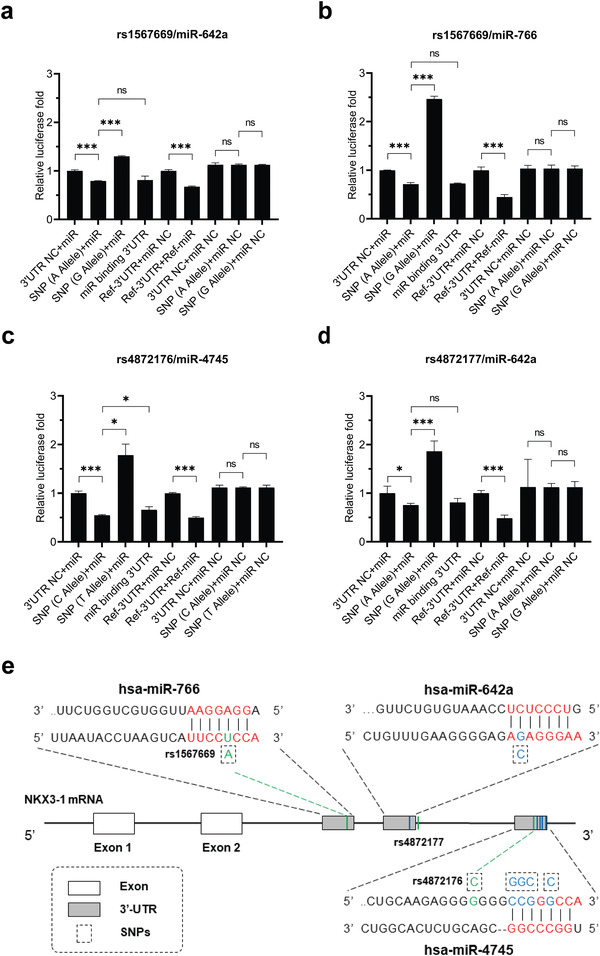
Dual‐luciferase reporter assays demonstrated the relationship between the genome‐wide significant 3′‐UTR SNPs and predicted miRNAs. a–d) Cotransfection of predicted miRNAs and luciferase reporters with the risk alleles of 3′‐UTR SNPs showed significantly lower luciferase activity than those with wild‐type alleles (the second and third bars). Cotransfection of hsa‐miR‐146b with TRAF6‐3′‐UTR was used as a positive control, which verified the efficiency of transfection system (the fifth and sixth bars). The luciferase activity without miRNA (with miR NC) was used as a negative control, which showed the effectiveness of miRNA cotransfection (the seventh, eighth, and ninth bars). *, *p* < 0.05; ***, *p* < 0.001 (Student's *t*‐test). e) The three predicted miRNA‐binding sites within the 3′‐UTR of NKX3‐1 mRNA. Nucleotides of the three genome‐wide significant 3′‐UTR SNPs are shown in green, and the SNPs in linkage disequilibrium region are shown in blue (LD, *R* ^2^ > 0.80). Abbreviations: SNP, single nucleotide polymorphism; miR, microRNA; ns, not significant; NC, negative control; Ref‐3′‐UTR: TRAF6‐3′‐UTR; Ref‐miR: hsa‐miR‐146b.

To further evaluate whether the association between these SNPs and *NKX3‐1* expression is regulated by miRNA, we predicted the interactions between the SNPs and miRNA using the MirSNP database.^[^ ^15^ ^]^ We found that these SNPs were also located in the binding sites of miR‐642a (rs1567669 and rs4872177), miR‐766 (rs1567669), and miR‐4745 (rs4872176) and might be associated with the binding affinity of miRNAs to these regions. Dual‐luciferase reporter assays were then applied to test whether the variants would influence the affinity of miRNA in these regions. The results showed that the G allele of rs1567669 significantly reduced the affinity of miR‐642a compared to the A allele (risk allele, *P* < 0.001, **Figure** **3**a). This led to a higher affinity for miR‐642a in the rs1567669 region when carrying the risk allele of this SNP (A allele); therefore, the expression of the reporter gene was downregulated (Figure 3a). Similarly, the risk alleles of other SNPs would also increase the affinity of the targeted miRNA and downregulate the expression of the reporter gene (Figure 3b–d). The three predicted miRNA‐binding sites within the 3′‐UTR of NKX3‐1 mRNA were also shown (Figure 3e).

**Figure 4:**
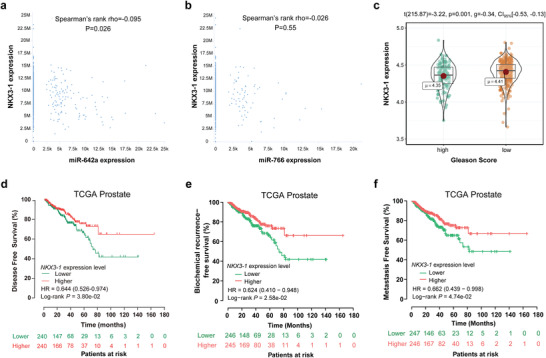
Bioinformatics analyses in TCGA cohort (*N* = 491). a,b) Scatter plots showing negative correlations between miR‐642a and *NKX3‐1* expression in PCa specimens (*P* = 0.026), but no significant association was observed between miR‐766 and *NKX3‐1* expression (*P* = 0.55). c) Higher *NKX3‐1* expression is associated with lower Gleason score (ISUP group < 5, *P* = 0.001). d–f) Kaplan–Meier curves depicting the disease‐, biochemical recurrence‐, and metastasis‐free survival of prostate cancer patients with high and low expression of *NKX3‐1* (all *P* < 0.05).

To evaluate the effect of the miRNAs on the expression of *NKX3‐1*, bioinformatic analyses based on The Cancer Genome Atlas (TCGA) database were performed. Overexpression of miR‐642a was significantly associated with low expression of *NKX3‐1* (Spearman's rank rho = −0.095, *P* = 0.026, **Figure** **4**a). However, no significant correlation between miR‐766 and *NKX3‐1* was observed (Spearman's rank rho = −0.026, *P* = 0.55, Figure 4b). Expression data of miR‐4745 were not available in the TCGA dataset. Additional analyses showed that higher expression of *NKX3‐1* was significantly associated with a lower Gleason score (International Society of Urological Pathology [ISUP] Group 5, *P* = 0.001, Figure 4c), better disease‐free survival of PCa (hazard ratio, HR = 0.64, 95% confidence interval, 95%CI: 0.53–0.97, *P* = 0.038, Figure 4d), better biochemical recurrence‐free survival (HR = 0.62, 95%CI: 0.41–0.95, *P* = 0.026, Figure 4e), and better metastasis free survival (HR = 0.66, 95%CI: 0.44–1.00, *P* = 0.047, Figure 4f). Similar results were found in multiple independent cohorts (Figures S3 and S4, Supporting Information). No significant association between miR‐642a or miR‐766 and disease prognosis was observed (both *P* > 0.05, Figure S5, Supporting Information), probably due to the relatively mild effect of miRNAs in this situation.

## 3 Discussion

A large number of disease‐associated SNPs have been identified in the past decade. Most of these variants are located in the intergenic region or intronic region. Their biological functions are poorly understood. Epigenetic regulation has been considered one of the critical pathways and bridges between genetic variants and RNA/protein expression. In the present study, we investigated whether germline common variants would interact with miRNA, thereby regulating the targeted genes and causing disease. To the best of our knowledge, this is the first genome‐wide 3′‐UTR SNP association study for PCa in Chinese population. We evaluated the association between 33 117 SNPs in 3′‐UTRs and PCa and found that rs4872176, rs4872177, and rs1567669 located in 8p21.2 (in the same LD region) were significantly associated with PCa. Additional bioinformatic analyses and functional studies suggested that the risk allele of rs1567669 could increase the affinity of miR‐642a (in the binding area of miR‐642a) in the 3′‐UTR of *NKX3‐1*. Given that rs1567669 and miR‐642a were significantly associated with the expression of *NKX3‐1*, the results indicated that rs1567669 might regulate *NKX3‐1* expression by influencing the binding of miR‐642a.

Variants at 8p21.2 were reported to be significantly associated with PCa in Caucasians.^[^ ^16^ ^]^ Our previous two‐stage confirmation association study based on a Chinese population also suggested that SNPs in this region were significantly associated with PCa.^[^ ^17^ ^]^ Aside from the association between the variants and gene expression based on eQTL results, the mechanism of gene regulatory control in this region is unknown. More importantly, the index SNPs (the most significant SNPs) from GWASs were usually not functional variants. Causal variants are often located in the LD region of the index SNPs. In the present study, the identified 3′‐UTR SNPs were located in the LD region of the reported SNPs (*R* ^2^ = 0.92, Figure S2, Supporting Information).^[^ ^17^ ^]^ Therefore, we suggested that rs1567669 could be a causal and functional variant causing PCa. Other variants in this region were found to be associated with the 5′‐UTR of *NKX3‐1*, which might regulate the expression of the gene via other biological mechanisms.^[^ ^18^ ^]^ These results also suggest a complex regulation of *NKX3‐1* by germline variants and indicate the significance of the variants in this region.

*NKX3‐1* is an androgen‐regulated homeobox gene and is highly expressed in prostate tissue.^[^ ^19^ ^]^ It is closely correlated with the cell growth and differentiation of prostate tissue.^[^ ^20^ ^]^ Loss of heterozygosity in the 8p21 region involving *NKX3‐1* was commonly reported in PCa tissue.^[^ ^19^, ^21^, ^22^ ^]^ Epigenetic regulation of *NKX3‐1* transcription and posttranslational modification of *NKX3‐1*, including phosphorylation and ubiquitination, were also reported.^[^ ^18^, ^23^, ^24^ ^]^ These reports suggest that *NKX3‐1* might serve as a tumor suppressor in PCa. In CPGEA cohort and other independent cohorts, *NKX3‐1* is found to consistently indicate higher expression in prostate tumor than normal (Figure S6, Supporting Information). This is likely because that *NKX3‐1* is regulated by androgen signaling, and the androgen receptor signaling pathway is usually hyperactivated in PCa. On the other hand, *NKX3‐1* shows invariably downregulated in the metastatic PCa (Figure S7, Supporting Information), suggesting that *NKX3‐1* is likely to be a suppressor. The downregulation of *NKX3‐1* in metastatic PCa could be explained by the fact that metastatic PCa is less likely to be sustained or driven by the androgen signaling. The results from the present study also suggest that the risk alleles of the SNPs were significantly correlated with the low expression of *NKX3‐1*, which was consistent with previous findings. Our results also provide a possible explanation for the biological mechanisms of carcinogenesis via germline genetic variants through regulating miRNA binding, hence adding additional evidence of a complex regulatory network targeting the tumor suppressor *NKX3‐1*.

As the largest genome‐wide 3′‐UTR SNP association study for PCa in the Chinese population, we provide a novel approach to explain the function of disease risk‐associated SNPs. Germline variants for cancer predisposition are usually different in races or ethnicities; however, our results provide an example that their biological mechanisms might be similar. In addition to the current indirect evaluation via dual‐luciferase reporter assays and bioinformatic analyses, direct evaluation of the 3′‐UTR SNP‐induced miRNA binding gene regulation should be performed in the future to further reveal the causal relationship.

## 4 Experimental Section

### Study Population

This three‐stage GWAS cohort with more than 5500 individuals was a part of the ChinaPCa Consortium (the Chinese Consortium for Prostate Cancer Genetics).^[^ ^6^ ^]^ A total of 1417 PCa cases and 1008 healthy population controls were enrolled in Stage 1. Detailed information on the study population of Stage 1 was described elsewhere.^[^ ^6^ ^]^ Stage 2 and Stage 3 were conducted based on a prospective, observational PCa biopsy study (Stage 2 and Stage 3: from August 2013 to November 2019; randomly grouped). Briefly, the study population met the criteria of prostate biopsy based on the clinical guidelines and received biopsy in three tertiary medical centers (Shanghai Ruijin Hospital, Shanghai Huashan Hospital, and Shanghai Cancer Center) in Shanghai, China.^[^ ^11^ ^]^ A total of 657 cases and 838 controls were enrolled in Stage 2, and 703 cases and 892 controls were enrolled in Stage 3. The study was approved by the institutional review board of each hospital, and written informed consent was obtained from each participant.

### Genotyping and Quality Control

All the participants were genotyped using the Illumina Human OmniExpress BeadChip (Stage 1) and the Illumina Asian Screening Array (Stages 2 and 3) with 731 458 SNPs and 659 184 SNPs, respectively. Samples were removed if they i) had an overall genotyping rate of < 95%; ii) had ambiguous gender; or iii) were duplicates or showed familial relationships (PI_HAT > 0.025). SNPs were excluded if they had i) a call rate of < 95%, ii) a minor allele frequency (MAF) of < 0.05, or iii) *P* < 1 × 10^−3^ in a Hardy–Weinberg equilibrium test among controls.

### Imputation and Quality Control

Ungenotyped SNPs were imputed using Minimac4 software^[^ ^25^ ^]^ with the East Asian population in 1000 Genomes Phase 3 serving as the reference haplotype. Imputed SNPs were excluded if they had i) an *r* ^2^‐value ( =imputation quality) < 0.3, ii) a call rate of < 95%, iii) an MAF of < 0.05, or iv) *P* < 1 × 10^−3^ in a Hardy–Weinberg equilibrium test in controls.

### Extracting 3′‐UTR miRNA Binding Site SNPs

MiRNA‐binding site SNPs in 3′‐UTRs were obtained from the MirSNP database (http://bioinfo.bjmu.edu.cn/mirsnp/search/).^[^ ^15^ ^]^ A total of 33 117 SNPs in 3′‐UTRs were analyzed in the ChinaPCa case‐control population (1417 cases and 1008 controls).

### eQTL Analysis and Bioinformatic Analysis

Cis‐eQTL analyses were performed using the GTEx project portal^[^ ^13^ ^]^ in different tissue samples. Significant results from any tissue were further evaluated. A confirmation of cis‐eQTL analysis was also performed in the normal prostate and tumor samples of the CPGEA.^[^ ^14^ ^]^ The association between miRNA expression and targeted gene expression was evaluated using TCGA in the UCSC Xena database.^[^ ^26^ ^]^ After eliminating patients with missing information, a total of 493 PCa patients with normalized RNA‐sequencing data, miRNA expression quantification data, and clinical data were recruited from TCGA database for further analyses. The sequence and microarray data were also retrieved from the Gene Expression Omnibus (GEO) database^[^ ^27^ ^]^ (GSE8402, GSE21034, GSE6099, GSE62872, GSE3325, GSE35988, GSE6099), FHCRC cohort (GSE77930),^[^ ^28^ ^]^ MSKCC cohort (GSE21032),^[^ ^29^ ^]^ and Stockholm cohort^[^ ^30^ ^]^ to perform additional validation study and survival analysis.

### 3′‐UTR Dual‐Luciferase Analysis

A plasmid was constructed, and the constructed target fragments were confirmed by sequencing. The 293T cells (RRID: CVCL_0063) were transfected in 24‐well plates with 3′‐UTR firefly luciferase reporter vector (0.1 µg) and Renilla luciferase control vector (0.02 µg), together with X‐tremeGENE HP Transfection Reagent (Roche, Mannheim, Germany, 2 µL) in Opti‐MEM (Gibco, Grand Island, NY, USA, 100 µL) per well. Luciferase activities were measured 48 h posttransfection using the Dual‐Luciferase Reporter Assay System (E2910; Promega, Madison, WI, USA) as described by the manufacturer. Briefly, the cells were removed and lysed in Passive Lysis Buffer 1× (300 µL) at 4 ℃ for 20 min. After equilibrating to room temperature, cell lysate (40 µL) and Luciferase Assay Reagent (20 µL) were mixed into each well, and then firefly luminescence was measured. Renilla luminescence was further measured after adding Stop & Glo Reagent (20 µL) to each well. All reporter assays were repeated three times independently. To adjust the effect of transfection efficiency, the ratios of firefly/Renilla luminescence for each well were calculated and normalized to the control wells that had been treated consistently (with the same luciferase reporter vector). Finally, the relative response ratios were calculated from the normalized ratios.

### Statistical Analysis

A logistic regression model was used to evaluate the association between each SNP and PCa using PLINK software (version 1.90).^[^ ^31^ ^]^ Only SNPs that reached *P* < 0.05 in Stage 1 were further evaluated in the Stage 2 confirmation study. A meta‐analysis of Stage 1 and Stage 2 was then performed to identify candidate SNPs (*P* < 5 × 10^−5^) and confirmed in Stage 3. Finally, a meta‐analysis of the three stages was performed, and a *P*‐value < 1.51 × 10^−6^ (0.05/33 117) was considered genome‐wide statistically significant after Bonferroni correction for multiplicity. Afterward, a PheWAS was performed between 2264 disease endpoints in 176 899 individuals based on the FinnGen study cohort (Data freeze release 4, publicly available November 30, 2020).^[^ ^12^ ^]^ All the data were presented as the means ± standard deviation (SD). Gene expression was tested using Mann–Whitney *U* test between two groups or Kruskal–Wallis rank test across three groups. The relative luciferase response ratios were compared using Student's *t*‐test. Spearman's correlation coefficient was also used to perform correlation analysis. Kaplan–Meier curves were applied in all survival analyses. Log‐rank test was carried out to compare disease‐, biochemical recurrence‐, and metastasis‐free survival between different groups in clinical cohorts. All statistical analyses, including Manhattan Plot and Q‐Q Plot for GWAS, were performed using R software (version 4.0.0).^[^ ^32^ ^]^ A two‐tailed *P* < 0.05 was considered statistically significant.

## Conflict of Interest

The authors declare no conflict of interest.

## Supporting information

Supporting Information

## Data Availability

The data that support the findings of this study are available from the corresponding authors upon reasonable request.
