# Supplementary material for: Genome‐Wide 3′‐UTR Single Nucleotide Polymorphism Association Study Identifies Significant Prostate Cancer Risk‐Associated Functional Loci at 8p21.2 in Chinese Population
Source: Adv Sci (Weinh). 2022 Jun 17;9(23):2201420. doi: 10.1002/advs.202201420 (PMC9376745; doi:10.1002/advs.202201420)

ADVANCED SCIENCE
Open Access

Supporting Information

for *Adv. Sci.*, DOI 10.1002/advs.202201420

Genome-Wide 3′-UTR Single Nucleotide Polymorphism Association Study Identifies Significant Prostate Cancer Risk-Associated Functional Loci at 8p21.2 in Chinese Population

Ning Zhang, Da Huang, Guangliang Jiang, Siteng Chen, Xiaohao Ruan, Haitao Chen, Jingyi Huang, Ao Liu, Wenhui Zhang, Xiaoling Lin, Yishuo Wu, Qin Zhang, Jing Li, James Hok-Leung Tsu, Gong-Hong Wei* and Rong Na**

Supporting Information

Germline variants at 8p21.2 associated with increased prostate cancer risk downregulate NKX3-1 expression via increased miRNA binding: a genome-wide 3'-UTR single nucleotide polymorphism association study in Chinese population

Ning Zhang, Da Huang, Guangliang Jiang, Siteng Chen, Xiaohao Ruan, Haitao Chen, Jingyi Huang, Ao Liu, Wenhui Zhang, Xiaoling Lin, Yishuo Wu, Qin Zhang, Jing Li, James Hok-Leung Tsu, Gong-Hong Wei*, and Rong Na**

Table S1. Demographic characteristics of the study populations.
Table S2. Association between the risk alleles of these SNPs and NKX3-1 expression in different normal tissues based on the Genotype-Tissue Expression (GTEx) database.
Figure S1. Q-Q plot of Stage 1 genome-wide 3'-UTR SNPs association study. The observed *P* value of each SNP is sorted from the largest to the smallest and plotted against expected values from a theoretical Chi-square distribution (the inflation factor $\lambda = 1.069$).
Figure S2. Fine-mapping of the ±200kb region of the significant SNPs (3'-UTR of *NKX3-1*). LocusZoom plot with each SNP in the region: X-axis stands for genomic position in Chr8. Y-axis stands for the -$\log_{10}$P-value of each SNP from Stage 1 genome-wide 3'-UTR SNPs association study. Loci in red indicated high correlation with each other which located in the same LD region of significant SNPs in 3'-UTR of *NKX3-1*.
Figure S3. *NKX3-1* are significantly downregulated in advanced PCa. Higher expression of *NKX3-1* was significantly associated with lower a)-d) Gleason score, and e)-f) tumor stage in multiple independent cohorts (all $P < 0.05$).
Figure S4. Kaplan-Meier curves of biochemical recurrence-free survival in prostate cancer patients with high and low expression of *NKX3-1* in two independent cohorts (all $P < 0.05$).
Figure S5. Kaplan-Meier curves of disease-free survival in prostate cancer patients with high and low expression of miR-642a or miR-766 in The Cancer Genome Atlas (TCGA) database (N = 480, all $P < 0.05$).
Figure S6. *NKX3-1* expression was significantly elevated in prostate tumor than normal prostate tissues in a) CPGEA, b) TCGA cohort, and c)-d) two other independent cohorts (all $P < 0.05$).
Figure S7. *NKX3-1* expression was significantly decreased in metastatic group than primary PCa and normal prostate tissues in multiple independent cohorts (all $P < 0.05$).

Table S1. Demographic characteristics of the study populations.

Characteristics	Stage 1 (n=2425)	Stage 2 (n=1495)	Stage 3 (n=1595)	*P* value[a]
PCa vs. Controls	1417 vs. 1008	657 vs. 838	703 vs. 892	0.943
Age, Median (IQR)	68 (61-75)	68 (62-75)	68 (62-74)	0.315
tPSA, Median (IQR)	24.0 (11.7-77.8)	13.8 (7.6-27.2)	12.5 (7.8-25.1)	0.232
GS ≥8, n/N (%)	537/1417 (37.9)	219/485 (45.2)	247/564 (43.8)	0.760

Abbreviation: PCa, prostate cancer; IQR, interquartile range; PSA, prostate-specific antigen; csPCa, clinically significant prostate cancer; GS, Gleason score.

[a] Baseline characteristics between Stage 2 and 3 were compared by Mann-Whitney U test for continuous variables and Chi-square test for categorical variables.

Table S2. Association between the risk alleles of these SNPs and NKX3-1 expression in different normal tissues based on the Genotype-Tissue Expression (GTEx) database.

Gencode Id	Gene Symbol	Variant Id	SNP Id	P-Value	NES	Tissue
rs1567669 eQTLs (P<0.0001)						
ENSG00000167034.9	NKX3-1	chr8_23681020_A_G_b38	rs1567669	1.30E-16	0.24	Muscle - Skeletal
ENSG00000167034.9	NKX3-1	chr8_23681020_A_G_b38	rs1567669	2.20E-09	0.32	Heart - Left Ventricle
ENSG00000167034.9	NKX3-1	chr8_23681020_A_G_b38	rs1567669	7.10E-07	0.25	Heart - Atrial Appendage
ENSG00000167034.9	NKX3-1	chr8_23681020_A_G_b38	rs1567669	5.00E-06	0.26	Adrenal Gland
ENSG00000167034.9	NKX3-1	chr8_23681020_A_G_b38	rs1567669	1.60E-05	0.42	Brain - Putamen (basal ganglia)
ENSG00000167034.9	NKX3-1	chr8_23681020_A_G_b38	rs1567669	1.60E-05	0.082	Testis
ENSG00000167034.9	NKX3-1	chr8_23681020_A_G_b38	rs1567669	2.00E-05	0.28	Liver
ENSG00000167034.9	NKX3-1	chr8_23681020_A_G_b38	rs1567669	2.00E-05	0.2	Esophagus - Mucosa
Meta-analysis of 49 tissues	NKX3-1	chr8_23681020_A_G_b38	rs1567669	9.94E-63		
rs4872176 eQTLs (P<0.0001)						
ENSG00000167034.9	NKX3-1	chr8_23680495_C_T_b38	rs4872176	2.60E-14	0.3	Nerve - Tibial
ENSG00000167034.9	NKX3-1	chr8_23680495_C_T_b38	rs4872176	6.60E-14	0.38	Heart - Left Ventricle
ENSG00000167034.9	NKX3-1	chr8_23680495_C_T_b38	rs4872176	1.30E-07	0.18	Cells - Cultured fibroblasts
ENSG00000167034.9	NKX3-1	chr8_23680495_C_T_b38	rs4872176	8.80E-07	0.31	Liver
ENSG00000167034.9	NKX3-1	chr8_23680495_C_T_b38	rs4872176	1.30E-06	0.43	Brain - Nucleus accumbens (basal ganglia)
ENSG00000167034.9	NKX3-1	chr8_23680495_C_T_b38	rs4872176	1.50E-06	0.14	Muscle - Skeletal
ENSG00000167034.9	NKX3-1	chr8_23680495_C_T_b38	rs4872176	4.90E-06	0.085	Testis
ENSG00000167034.9	NKX3-1	chr8_23680495_C_T_b38	rs4872176	2.50E-05	0.17	Adipose - Subcutaneous
ENSG00000167034.9	NKX3-1	chr8_23680495_C_T_b38	rs4872176	3.00E-05	0.33	Brain - Caudate (basal ganglia)
ENSG00000167034.9	NKX3-1	chr8_23680495_C_T_b38	rs4872176	8.60E-05	0.13	Adipose - Visceral (Omentum)
Meta-analysis of 49 tissues	NKX3-1	chr8_23681020_A_G_b38	rs4872176	2.50E-79		
rs4872177 eQTLs (P<0.0001)						
ENSG00000167034.9	NKX3-1	chr8_23680913_A_G_b38	rs4872177	2.00E-16	0.27	Muscle - Skeletal
ENSG00000167034.9	NKX3-1	chr8_23680913_A_G_b38	rs4872177	9.40E-10	0.33	Heart - Atrial Appendage
ENSG00000167034.9	NKX3-1	chr8_23680913_A_G_b38	rs4872177	1.10E-09	0.34	Heart - Left Ventricle
ENSG00000167034.9	NKX3-1	chr8_23680913_A_G_b38	rs4872177	7.90E-09	0.29	Esophagus - Mucosa

ENSG00000167034.9	NKX3-1	chr8_23680913_A_G_b38	rs4872177	5.90E-08	0.42	Liver
ENSG00000167034.9	NKX3-1	chr8_23680913_A_G_b38	rs4872177	1.00E-05	0.47	Brain - Putamen (basal ganglia)
ENSG00000167034.9	NKX3-1	chr8_23680913_A_G_b38	rs4872177	2.10E-05	0.2	Adipose - Subcutaneous
ENSG00000167034.9	NKX3-1	chr8_23680913_A_G_b38	rs4872177	2.50E-05	0.4	Brain - Caudate (basal ganglia)
ENSG00000167034.9	NKX3-1	chr8_23680913_A_G_b38	rs4872177	9.10E-05	0.18	Skin - Sun Exposed (Lower leg)
Meta-analysis of 49 tissues	NKX3-1	chr8_23681020_A_G_b38	rs4872177	4.01E-74		

Abbreviation: NES, normalized effect size.

Figure S1. Q-Q plot of Stage 1 genome-wide 3'-UTR SNPs association study. The observed *P* value of each SNP is sorted from the largest to the smallest and plotted against expected values from a theoretical Chi-square distribution (the inflation factor λ = 1.069).

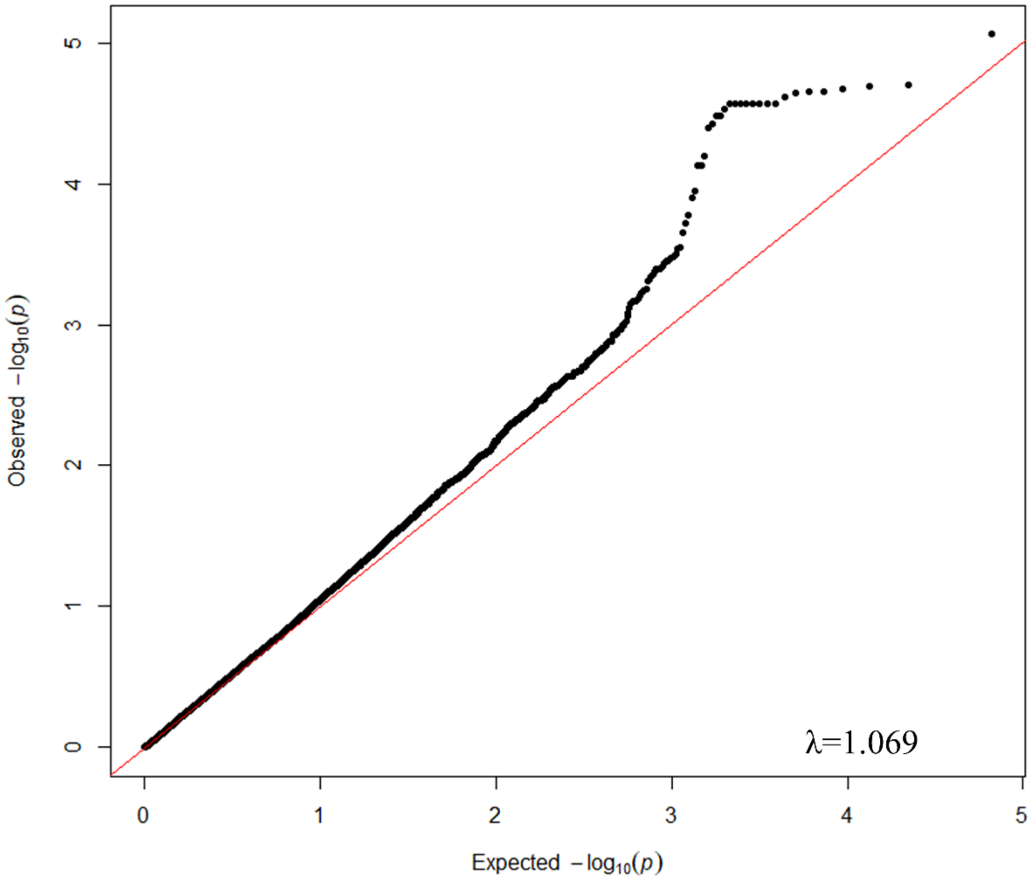

Figure S2. Fine-mapping of the ±200kb region of the significant SNPs (3’-UTR of *NKX3-1*). LocusZoom plot with each SNP in the region: X-axis stands for genomic position in Chr8. Y-axis stands for the -log$_{10}$P-value of each SNP from Stage 1 genome-wide 3’-UTR SNPs association study. Loci in red indicated high correlation with each other which located in the same LD region of significant SNPs in 3’-UTR of *NKX3-1*.

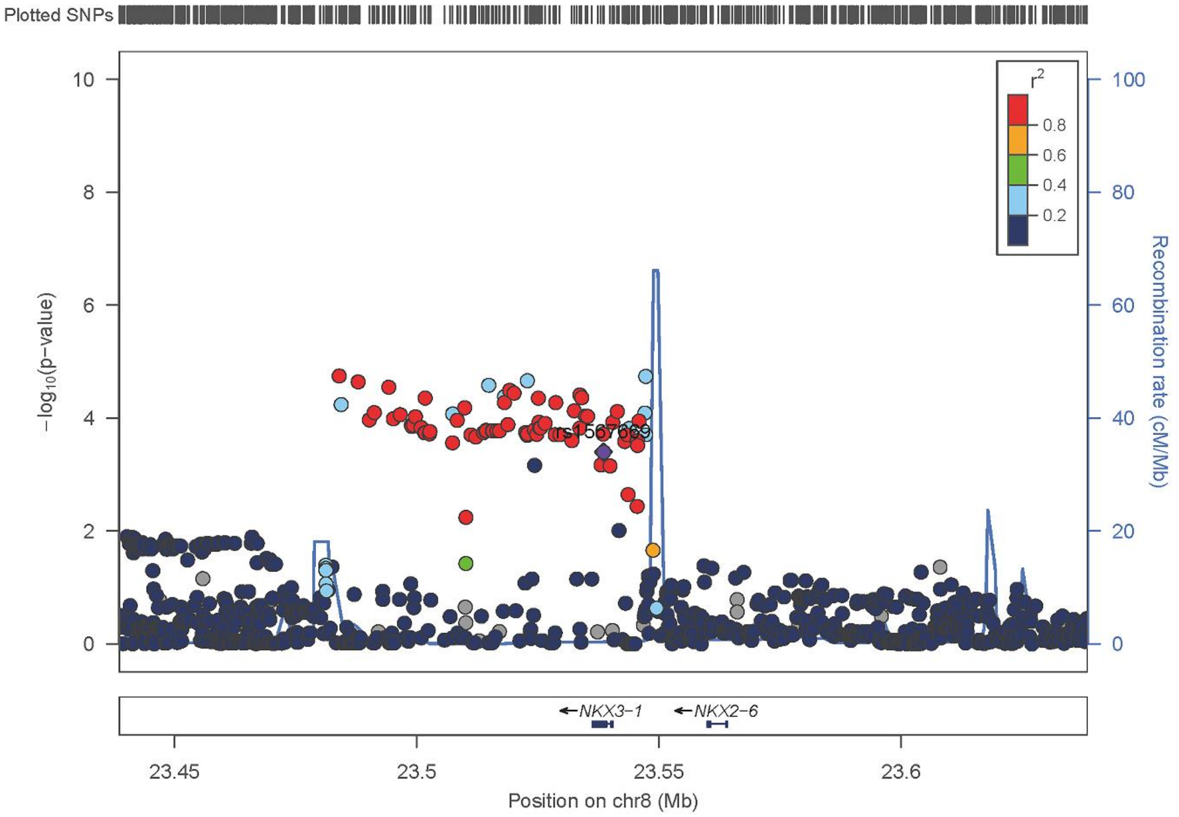

Figure S3. ***NKX3-1*** **are significantly downregulated in advanced PCa.** Higher expression of *NKX3-1* was significantly associated with lower a)-d) Gleason score, and e)-f) tumor stage in multiple independent cohorts (all *P* < 0.05).

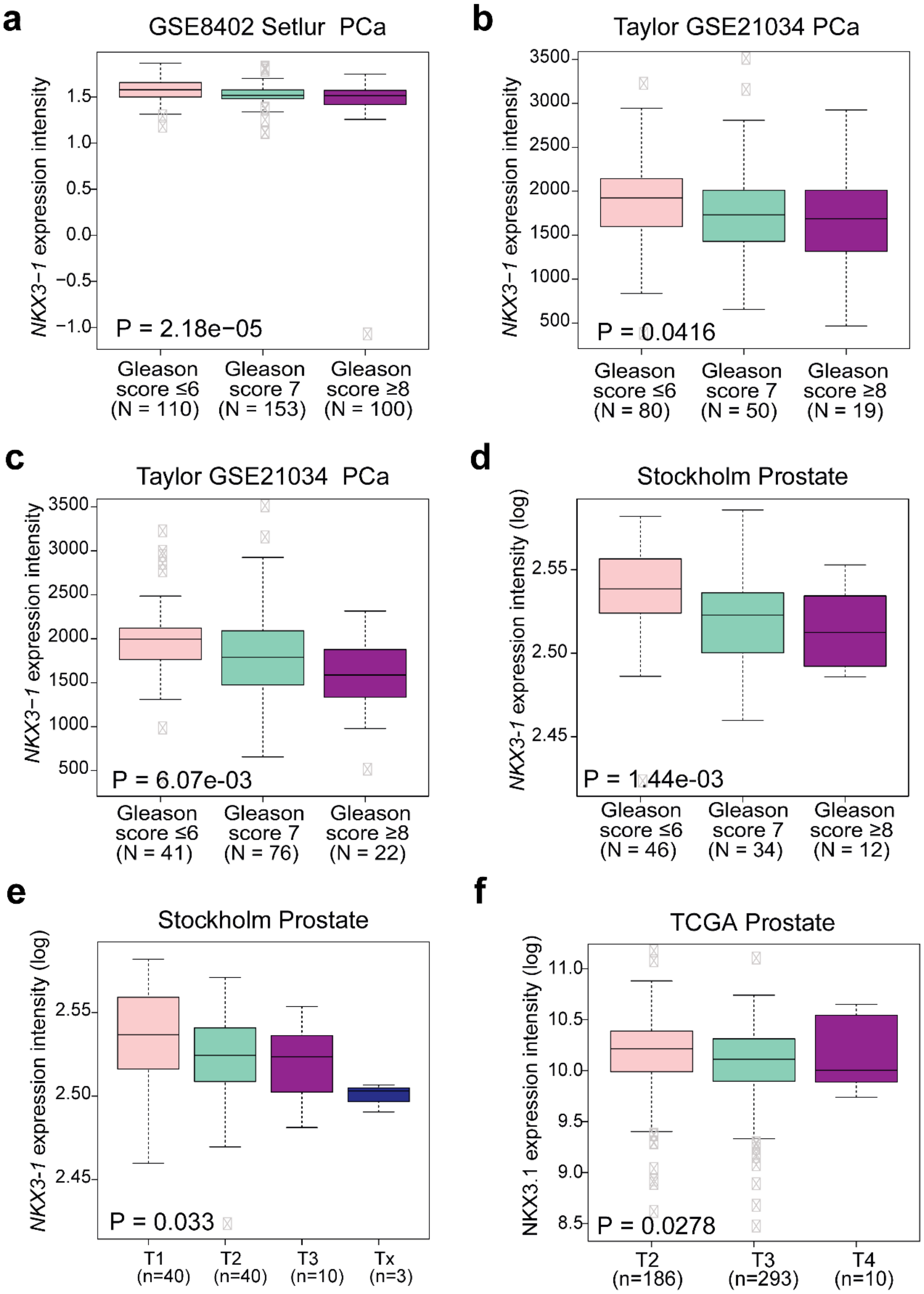

Figure S4. Kaplan-Meier curves of biochemical recurrence-free survival in prostate cancer patients with high and low expression of *NKX3-1* in two independent cohorts (all P < 0.05).

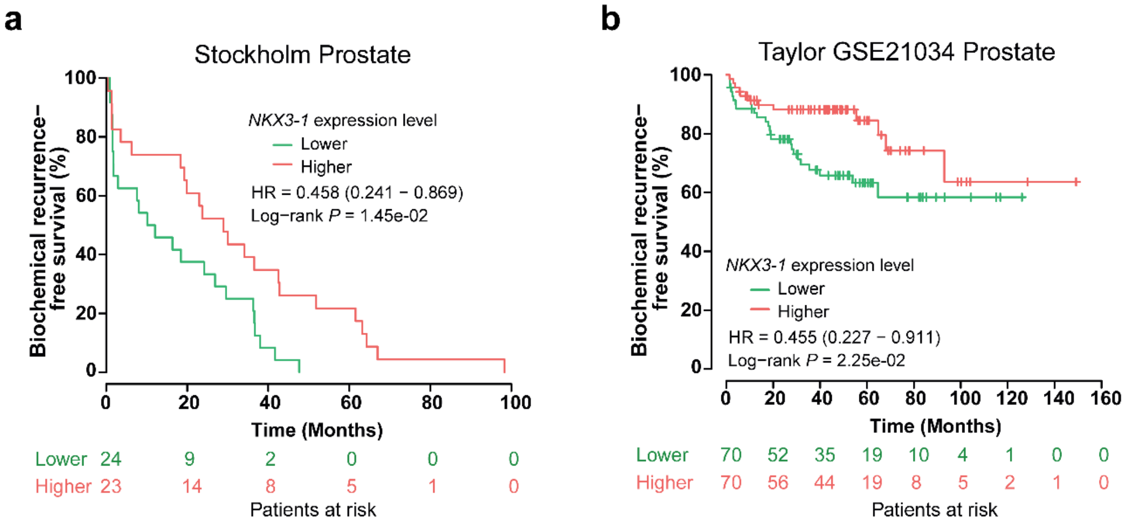

Figure S5. Kaplan-Meier curves of disease-free survival in prostate cancer patients with high and low expression of miR-642a or miR-766 in The Cancer Genome Atlas (TCGA) database (*N* = 480, all *P* < 0.05).

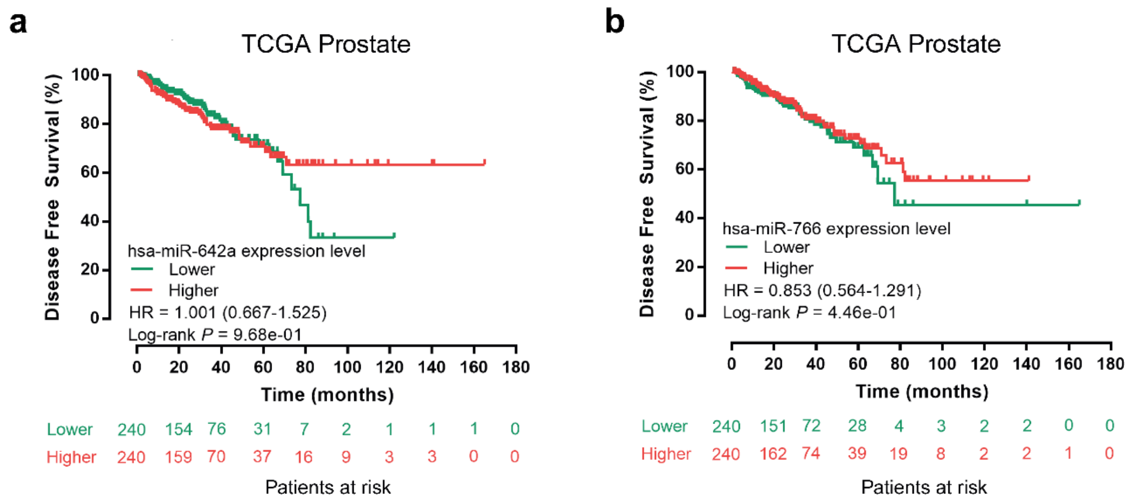

Figure S6. ***NKX3-1*** **expression was significantly elevated in prostate tumor than normal prostate tissues in a) CPGEA, b) TCGA cohort, and c)-d) two other independent cohorts (all** $P < 0.05$**).**

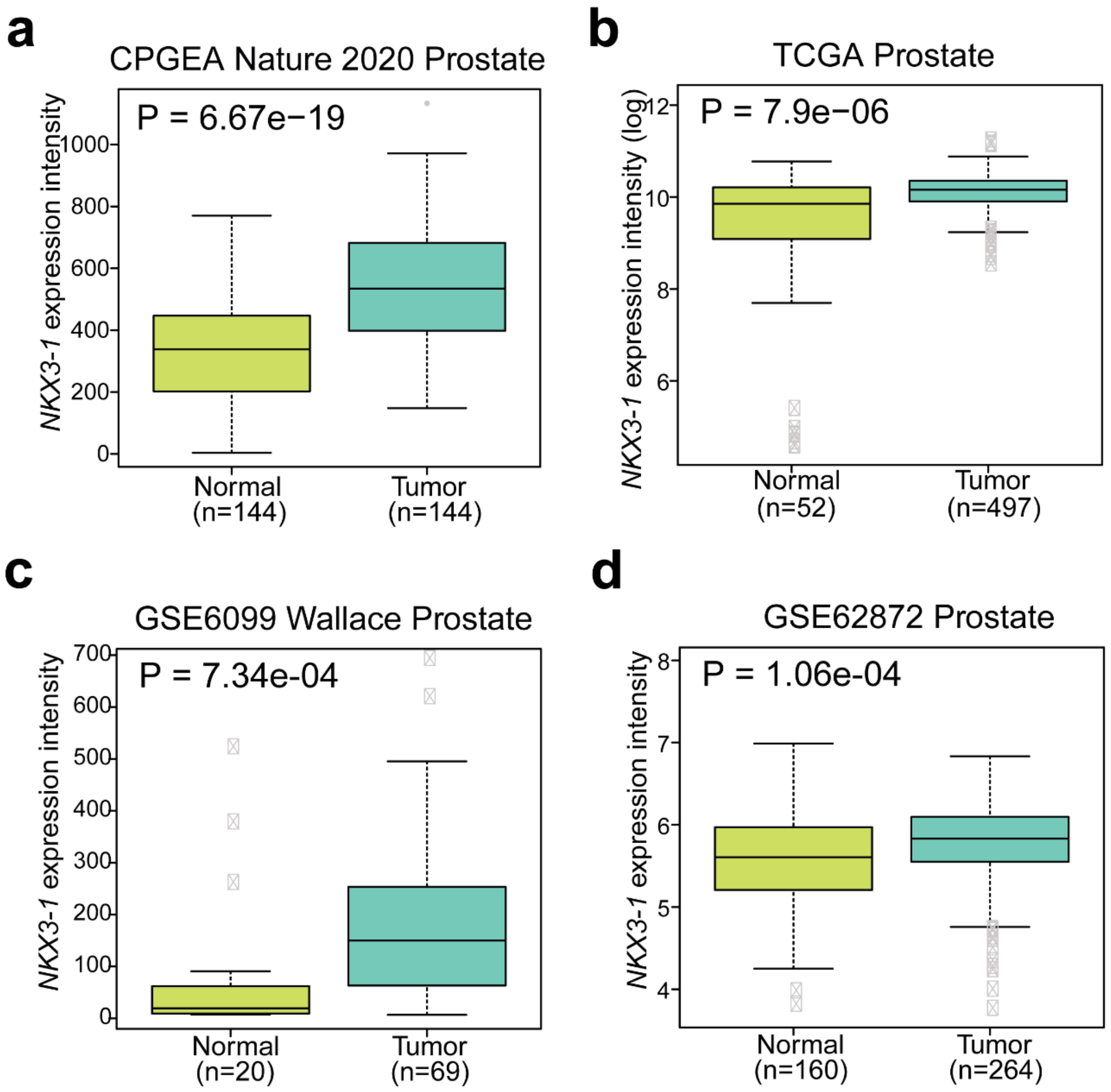

Figure S7. ***NKX3-1*** **expression was significantly decreased in metastatic group than primary PCa and normal prostate tissues in multiple independent cohorts (all *P* < 0.05).**

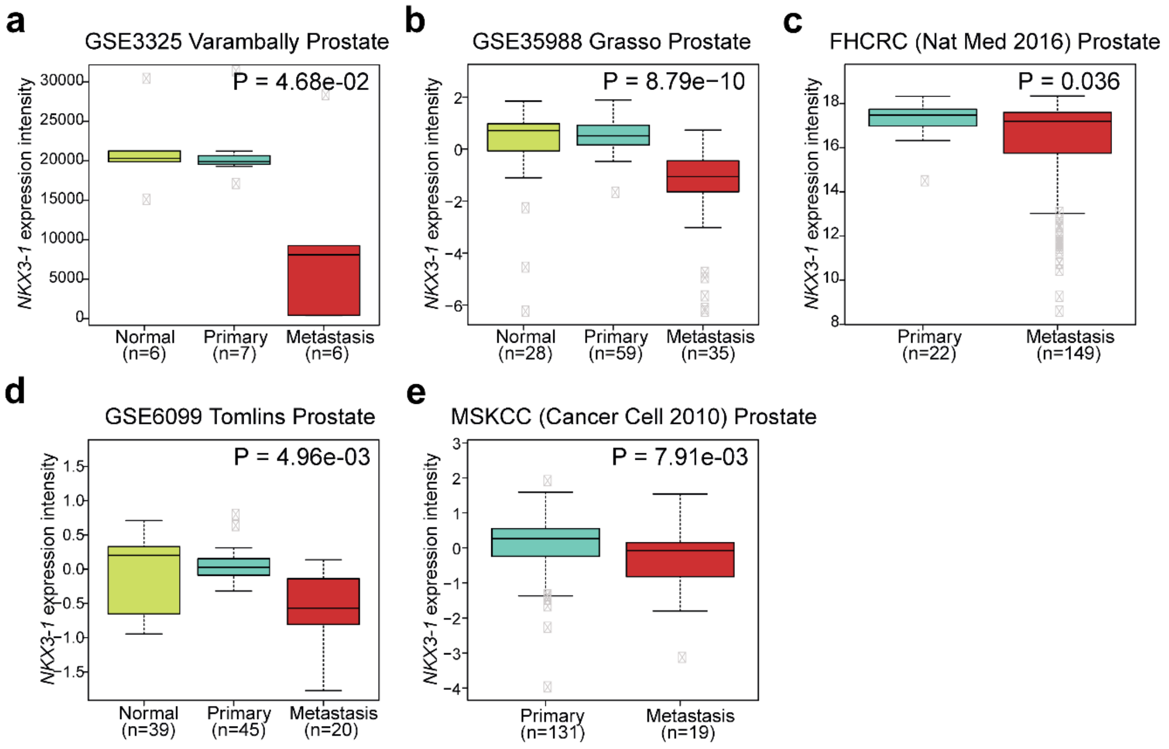

Supplement: Supplementary file 1 — Supporting Information [file ADVS-9-2201420-s001.pdf]
